# Nanoparticles in Drug Delivery: From History to Therapeutic Applications

**DOI:** 10.3390/nano12244494

**Published:** 2022-12-19

**Authors:** Obaid Afzal, Abdulmalik S. A. Altamimi, Muhammad Shahid Nadeem, Sami I. Alzarea, Waleed Hassan Almalki, Aqsa Tariq, Bismillah Mubeen, Bibi Nazia Murtaza, Saima Iftikhar, Naeem Riaz, Imran Kazmi

**Affiliations:** 1 Department of Pharmaceutical Chemistry, College of Pharmacy, Prince Sattam Bin Abdulaziz University, Al Kharj 11942, Saudi Arabia; 2 Department of Biochemistry, Faculty of Science, King Abdulaziz University, Jeddah 21589, Saudi Arabia; 3 Department of Pharmacology, College of Pharmacy, Jouf University, Sakaka 72341, Saudi Arabia; 4 Department of Pharmacology, College of Pharmacy, Umm Al-Qura University, Makkah 21955, Saudi Arabia; 5 Institute of Molecular Biology and Biotechnology (IMBB), The University of Lahore, Lahore 54000, Pakistan; 6 Department of Zoology, Abbottabad University of Science and Technology (AUST), Abbottabad 22310, Pakistan; 7 School of Biological Sciences, University of Punjab, Lahore 54000, Pakistan; 8 Department of Pharmacy, COMSATS University, Abbottabad 22020, Pakistan

**Keywords:** drug delivery, nanomedicine, therapeutics, nanoparticles, personalized medicine

## Abstract

Current research into the role of engineered nanoparticles in drug delivery systems (DDSs) for medical purposes has developed numerous fascinating nanocarriers. This paper reviews the various conventionally used and current used carriage system to deliver drugs. Due to numerous drawbacks of conventional DDSs, nanocarriers have gained immense interest. Nanocarriers like polymeric nanoparticles, mesoporous nanoparticles, nanomaterials, carbon nanotubes, dendrimers, liposomes, metallic nanoparticles, nanomedicine, and engineered nanomaterials are used as carriage systems for targeted delivery at specific sites of affected areas in the body. Nanomedicine has rapidly grown to treat certain diseases like brain cancer, lung cancer, breast cancer, cardiovascular diseases, and many others. These nanomedicines can improve drug bioavailability and drug absorption time, reduce release time, eliminate drug aggregation, and enhance drug solubility in the blood. Nanomedicine has introduced a new era for drug carriage by refining the therapeutic directories of the energetic pharmaceutical elements engineered within nanoparticles. In this context, the vital information on engineered nanoparticles was reviewed and conferred towards the role in drug carriage systems to treat many ailments. All these nanocarriers were tested in vitro and in vivo. In the coming years, nanomedicines can improve human health more effectively by adding more advanced techniques into the drug delivery system.

## 1. Introduction

Drug delivery systems (DDSs) have been used in past eras to treat numerous ailments. All medicines rely on pharmacologic active metabolites (drugs) to treat diseases [1]. Some of the drugs are designed as the inactive precursor, but they become active when transformed in the body [2]. Their effectiveness depends on the route of administration. In conventional drug delivery systems (CDDSs), drugs were delivered usually via oral, nasal, inhaled, mucosal, and shot methods [3]. The conventionally delivered drugs were absorbed less, distributed randomly, damaged unaffected areas, were excreted early, and took a prolonged time to cure the disease [4]. They were less effective due to many hurdles like their enzymatic degradation or disparity in pH, many mucosal barriers, and off-the-mark effects, and their immediate release enhanced toxicity in blood [5].

**Figure 1:**
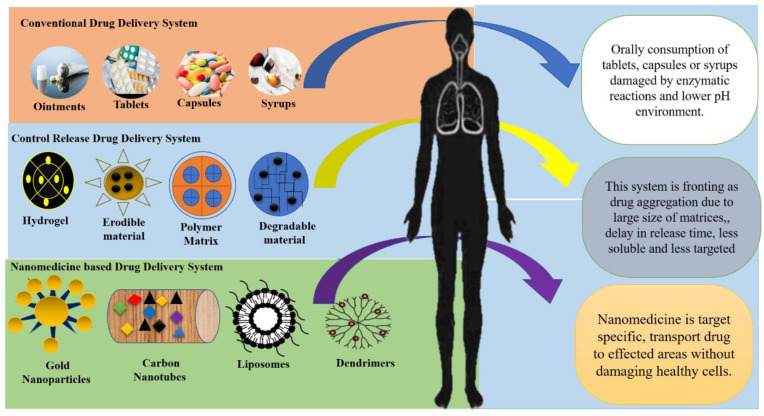
Illustration of how traditional medications were administered without the use of nanocarriers and harm was done to healthy organs or cells. In contrast, modern procedures use nanomedicines to transport medications to specific parts of the body.

Due to all such reasons, the controlled-release drug delivery system was developed. Such evolution in the DDS enhances drug effectiveness in many ways [6]. DDSs have been engineered in recent years to control drug release [7]. Such engineered DDSs used various novel strategies for controlled drug release into the diseased areas. These strategies were erodible material, degradable material, matrix, hydrogel, osmotic pump, and reservoir [8]. They all provided a medium for the medicines to deliver at the desired sites like tissues, cells, or organs. In these approaches, drugs are often available for many diseases [9]. Such strategies were unsuccessful due to lower distribution, less solubility, higher drug aggregation, less target selection, and poor effects for disease treatment [10]. Moreover, drug development is the most expensive, intricate, and time-consuming process [5]. The innovative drug findings involved the identification of new chemical entities (NCEs), [11] having the vital distinguishing characteristics of drug capacity and pharmaceutical chemistry. This methodology, however, was confirmed to be less effective in terms of the overall attainment percentage [12], as 40% of drug development was botched due to its changeable responses and unpredicted noxiousness in humans [13]. From past decades until now, drug development and its delivery are shifting from the micro to the nano level to prolong life expectancy by revolutionizing drug delivery systems (Figure 1) [14].

In 1959, Feynman was the first physicist to introduce the notion of nanotechnology in the lecture entitled “There’s Plenty of oom at the Bottom”. This concept initiated remarkable developments in the arena of nanotechnology [15]. Nanotechnology is the study of extremely tiny things and is basically the hub of all science disciplines including physics, chemistry, biology, engineering, information technology, electronics, and material science [16]. The structures measured with nanotechnology range from 1–100 nm at the nanoscale level [17]. Nanoparticles have different material characteristics because of submicroscopic size and also provide practical implementations in a wide range of fields including engineering, drug delivery, nanomedicine, environmental indemnification, and catalysis, as well as target diseases such as melanoma and cardiovascular diseases (CVD), skin diseases, liver diseases, and many others [18].

Therefore, medicines linked with nanotechnology can enhance efficiency of medicines and their bioavailability [19]. The relation of nanoparticles to biomedicine was demonstrated in late the 1970s, and over 10,000 publications have referred to this association with the term “nanomedicine”. Almost thirty papers on this term were accessible by 2005 [20].

After 10 to 12 years, Web of Science published more than 1000 nanomedicine articles in 2015 and most of the articles relating nanoparticles (NPs) for biomedical usage [21]. Nanocarriers such as dendrimers, liposomes, peptide-based nanoparticles, carbon nano tubes, quantum dots, polymer-based nanoparticles, inorganic vectors, lipid-based nanoparticles, hybrid NPs, and metal nanoparticles are the advanced forms of NPs [22]. Nanoparticles are nowadays a growing arena for drug delivery, microfluidics, biosensors, microarrays, and tissue micro-engineering for the specialized treatment of diseases [23,24,25].

Nanoparticles are less effective and can treat cancer by selectively killing all cancerous cells [26]. In 2015, the Food and Drug Administration (FDA) approved the clinical trials of onivyde nanomedicine in the treatment of cancer [27]. The characteristic properties of nanocarriers are physicochemical properties, supporting the drugs by improving solubility, degradation, clearance, targeting, theranostics, and combination therapy [28]. Studies on nanomedicine based on protein used for drug delivery in which various protein subunits combine to deliver medicine on site to a specific tumor have been reported [29]. Many altered kinds and forms of nanocarriers arranged to carry medicine are protein-based podiums, counting several protein coops, nanoparticles, hydrogels, films, microspheres, tiny rods, and minipellets [30]. All proteins, including ferritin–protein coop, the small heat shock protein (sHsp) cage, plant-derived viral capsids, albumin, soy and whey protein, collagen, and gelatin-implemented proteins are characterized for drug carriage [31].

**Figure 2:**
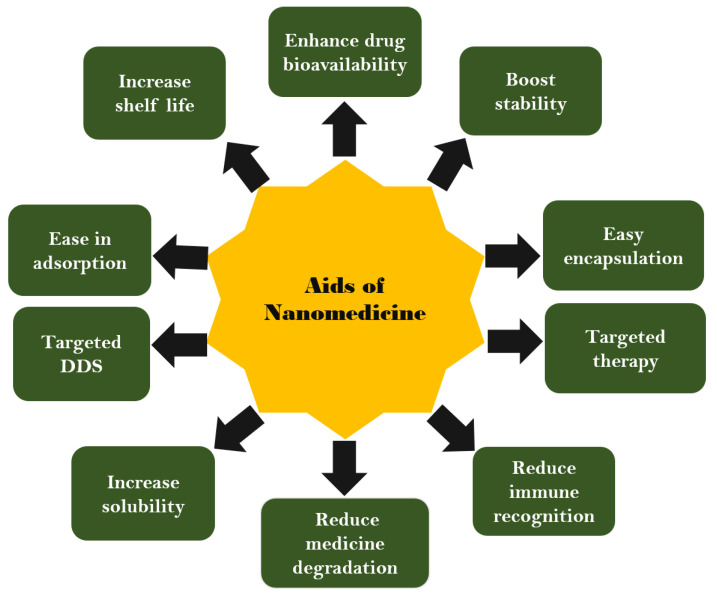
Aids of using nanomedicine platform for delivering drugs to the tumor complex.

The nanomedicines are escorted in a new-fangled epoch, meant for drug carriage by refining the therapeutic directories of the energetic pharmacological elements engineered inside nanoparticles [32]. In this epoch, nanomedicine-based targeted-design structures can deliver multipurpose freight with favorable pharmacokinetics and capitalized so as to enhance drug specificity, usefulness, and safety, as shown in (Figure 2) [33]. The failure of chemotherapeutic approaches has increased the recurrence chances of disease, which enhances the complexity of lethal diseases [34].

## 2. History

Petros and his colleague reported a study about mid-19th century work on nanotechnology. As they reported, polymers and drugs were conjugated in 1955 [35], the first controlled-release polymer device appeared in 1964, the liposome was discovered by Bangham in 1965, albumin-based NPs were reported in 1972, liposome-based drugs were formulated in 1973, the first micelle was formulated and approved in 1983, the FDA approved the first controlled formulation in 1989, and first polyethylene glycol (PEG) conjugated with protein entered the market in 1990 [36]. Further studies have produced incredibly encouraging results for treating a variety of disorders (Table 1).

## 3. Recent Approaches Used in Drug Carriage System for Treatment of Various Diseases

### 3.1. Brain Drug Delivery System and Its Types

Under the most pathological circumstances of diseases such as strokes, seizures, multiple sclerosis, AIDS, diabetes, glioma, Alzheimer’s disease, and Parkinson’s disease, the blood–brain barrier (BBB) is disrupted [103]. An important reason for the breakdown of the blood–brain barrier is the remodeling of the protein complex in intra-endothelial junctions under the pathological conditions [104]. Normally, the blood–brain barrier acts to maintain blood–brain homeostasis by preventing entry of macromolecules and micromolecules from the blood [105]. If a drug crosses the BBB, it restricts accumulation of the drug in the intracerebral region of brain, and bioavailability is reduced, due to which brain diseases cannot be treated [106]. Therefore, the optimal drug delivery system (DDS) is a cell membrane DDS, virus-based DDS, or exosome-based DDS designed for BBB penetrability, lesion-targeting ability, and standard safety [107]. For the cure of brain diseases, the nanocarrier-assisted intranasal drug carriage system is widely used [108]. Now, at the advanced level, drugs poorly distributed to the brain can be loaded into a nanocarrier-based system, which would interact well with the endothelial micro vessel cells at the BBB and nasal mucosa to increase drug absorption time and the olfactory nerve fibers to stimulate straight nose-to-brain delivery [109], thus greater drug absorption in brain parenchyma through the secondary nose-to-blood-to-brain pathway [110]. The current strategies used are viral vectors, nanoparticles, exosomes, brain permeability enhancers, delivery through active transporters in the BBB, alteration of administration route, nanoparticles for the brain, and imaging/diagnostics under diseased conditions [111].

#### 3.1.1. Role of Nanocarriers in Alzheimer’s Disease

Alzheimer’s disease is one of the fastest growing neurodegenerative diseases in the elderly population. Clinically, it is categorized by abstraction, damage to verbal access, and diminishing in spatial skills and reasoning [112]. Furthermore, engrossment of amyloid β (Aβ) aggregation and anxiety in the brain have significant parts [113]. The treatment of different diseases with nanotechnology-based drug delivery uses nanotechnology-based approaches [114]. In Alzheimer’s diseases, polymeric nanoparticles, liposomes, solid lipid nanoparticles, nano-emulsions, micro-emulsions, and liquid-crystals are used for treatment.

##### Polymeric Nanoparticles

I. The drug *Tacrine* was loaded on polymeric nanoparticles and administered through an intravenous route. It enhanced the concentration of tacrine inside the brain and also reduced the whole-dose quantity [115].
II. *Rivastigmine* drug was loaded on polymeric nanoparticles and administered through an intravenous route. It enhanced learning and memory capacities [116].

##### Solid Lipid Nanoparticles (SLNPs)

SLNPs enhanced drug retention in the brain area, raising absorption across the BBB [117]. Some of the drug’s effects are listed below.

I. *Piperine* drug is loaded on solid lipid nanoparticles through an intraperitoneal route inside the brain to decrease plaques and masses and to increase AChE enzyme activity [118].
II. *Huperzine A* improved cognitive functions. No main irritation was detected in rat skin when the drug was loaded on SLNPs in an in vitro study [119].

In recent reports, the coating of SLNPs with polysorbate enhances drug bioavailability [120,121]. Some of the coated NPs are listed below.

I. The drug clozapine was loaded on a Dynasan 116 [Tripalmitin] lipid matrix coated with surfactant Poloxamer 188, Epikuron 200 to unload the drug safely into the brain microenvironment [122,123].
II. Vitamin A was loaded on a lipid matrix Glyceryl behenate with coated surfactant hydroxypropyl distarch to unload the drug safely across the BBB [124,125].
III. Diminazine was loaded on a stearic acid matrix coated with polysorbate 80 to deliver to an infected area safely [126,127].
IV. Doxorubicin was loaded on stearic acid SLNs coated with Taurodeoxycholate surfactant to deliver the drug without reducing its effectiveness [128,129].

##### Liposomes

Liposomes have gained attention as auspicious tactics for brain-targeted drug delivery [130]. The recorded beneficial features of liposomes are their capacity to integrate and carry a large quantity of drugs and their likelihood to adorn their exterior with diverse ligands [131,132].

- *Curcumin–PEG derivative* was loaded on liposomes and showed high affinity on senile plaques in an ex vivo experiment. Furthermore, in vitro it demonstrated the ability for Aβ aggregation and was taken inside by the BBB in a rat model [133].
- *Folic acid* was loaded on liposomes, administered through an intranasal route and absorbed through the nasal cavity [134].

##### Nanoemulsions

I. *Beta-Asarone* was loaded on nanoemulsions, administered through an intranasal route, and enhanced bioavailability [130].

##### Micro Emulsion

I. *Tacrine* was loaded on a microemulsion and improved memory. Such nanoparticles absorbed rapidly via the nose to the brain through an intranasal route [135].

##### Liquid Crystals

I. *T. divaricate* was loaded on liquid crystals and injected through a transdermal route. It increased permanency of the drug in designs and also increased skin infusion and retention [136].

#### 3.1.2. Role of Nanocarriers in Parkinson’s Disease (PD)

Parkinson’s disease is considered the second most common neurological ailment, and it faces problems in reliable drug delivery for treatment and diagnosis [137]. The conventional anti-Parkinson’s drug is *Levodopa*, but it experiences low bioavailability and deprived transfer to the brain; this is the most thought-provoking problem [138]. To solve this problem, nanotechnology comes to the fore with insightful solutions to solve this problem. Various nanoparticles like metal nanoparticles, quantum dots, cerium oxide nanoparticles, organic nanoparticles, liposomes, and gene therapy are used in PD treatment [139]. All these nanoparticles enable drugs to enter through numerous ways across the blood–brain barrier (BBB) [140]. In the current study, Bhattamisra et al. reported *Rotigotine* drug loaded on chitosan NPs in human SH-SY5Y neuroblastoma cells and delivered from the nose to the brain in rat model of Parkinson’s disease. A study of the pharmacokinetic data proposed that the intranasal route is the best path for a straight channel of rotigotine to the brain [125].

##### Ropinirole (RP)

Ropinirole (RP) is a dopamine agonist used for Parkinson’s treatment. RP-loaded solid lipid nanoparticles (RP-SLNs) with nanostructured lipid carriers (RP-NLCs) comprising hydrogel (RP-SLN-C and RP-NLC-C) formulations are better for oral and topical distribution [141]. Generally, the results confirmed that lipid nanoparticles and consistent hydrogel formulations can be measured as another carriage methodology for the upgraded oral and topical delivery of RP for the active treatment of PD [142]. Neurodegenerative pathologies such as AD and PD can be treated with solid lipid nanoparticles, as this permits the drug to cross the BBB and reach the damaged area of the central nervous system [143].

### 3.2. Mechanism of Nanoparticles’ Brain Drug Delivery (across BBB)

**Figure 3:**
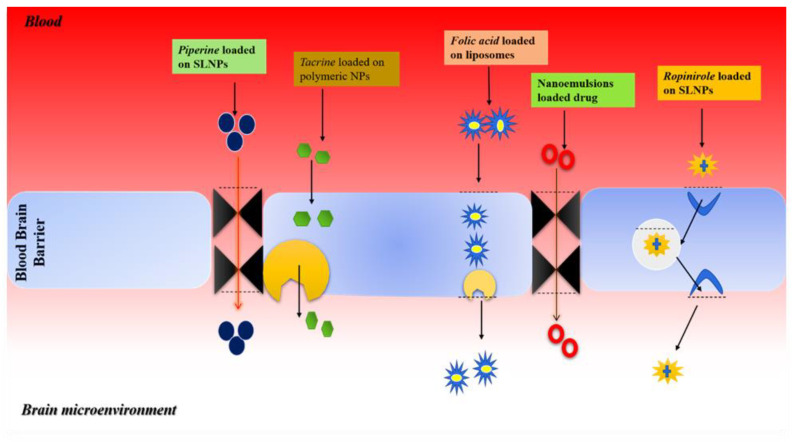
Diagram showing the mechanism of targeted drug delivery across BBB in brain microenvironment. Piperine loaded on SLNPs is injected intraperitonially, across BBB efferently to stop plaque formation. Polymeric nanoparticles are used for Tacrine delivery inside the brain, folic acid are loaded on the liposomes crossing blood–brain barrier to treat *Alzheimer’s* disease, while nanoemulsions and SLNP are loaded with drugs used to deliver medicines inside the targeted brain area to cure *Parkinson’s* disease.

The NPs are commonly administered via intranasal, intraventricular, intraparenchymal routes. All these routes enabled nanoparticles to cross the BBB due to their small size. When nanoparticles reach the BBB, several mechanisms are used, like receptor-mediated mechanisms, active transport, and passive transport to deliver nanoparticles into the brain. Nanoparticles are small in size, can diffuse passively across the endothelial cells of the BBB, and can interact favorably with brain receptors and recognize ligands for interaction (Figure 3) [144].

### 3.3. Advantages and Disadvantages of Nanomedicines

When employed for brain illnesses, nanomedicines have both benefits and drawbacks (Table 2).

## 4. Nanocarriers Role in Major Cancers

### 4.1. Brain Cancer

Brain malignancy is the most critical disease in the sense of treatment [150]. Malignancies of the brain are most difficult to treat due to limits imposed by the blood–brain barrier [151]. The brain microvascular endothelium is present in the BBB and creates barriers that distinguish blood from the neural tissues of the brain [152]. The BBB prevents the entry of harmful toxins, xenobiotic and other metabolites from entering the brain [153]. The majority of brain cancers include glioma and glioblastoma. Both of these are among the most lethal forms of brain cancer [154]. The annual occurrence is 5.26 per 100,000 people or 17,000 new diagnoses each year. The most common treatment is radiation surgery and chemotherapy, usually implemented with with temozolomide (TMZ) [155]. Nanoparticles have a high potential to treat brain cancer because of their small size in nm, tissue-specific targeting properties, and ease in crossing the BBB [156] (Table 3).

### 4.2. Breast Cancer

**Figure 4:**
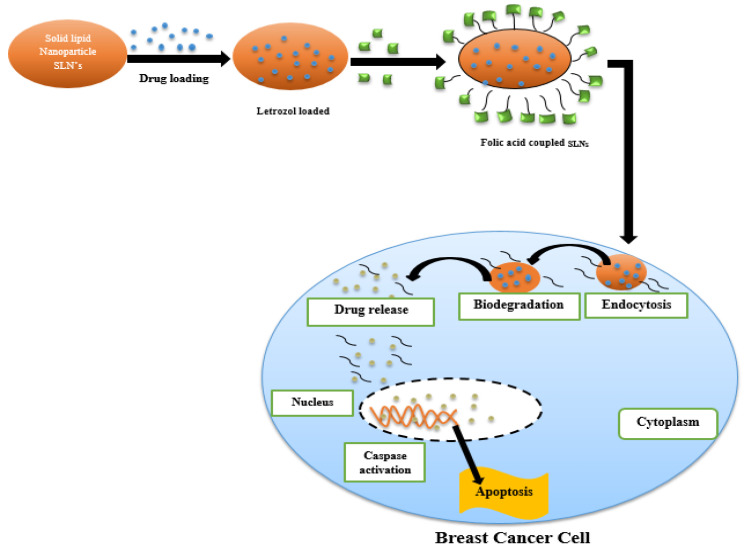
Schematic representation of mechanism of drug letrozol loaded on solid lipid nanoparticles (SLNs) and folic acid coupled to SLNs. The whole carrier was delivered inside the animal rat model to treat effects on breast cancer cell lines. Inside cytoplasm, biodegradation occurred, as well as drug release and caspases’ activation inside nucleus, causing apoptosis.

Cancer causes major deaths all over the world. Tumors spread due to the proliferation of cells [171], which invade through the lymphatic system to various parts of the body if they becomes malignant [172]. According to WHO, the ratio of deaths globally due to cancer is assessed to be 13%, attributing 8.2 million deaths every year [173]. Breast cancer is the most recorded type of melanoma present in only females, and its severity leads to mortality more often than lung cancer [174]. In 2012, estimated female breast cancer cases were 1.7 million, with 25% of deaths all over the world [175]. In a recent study, a report published in the name of Global Cancer Statistics 2020: GLOBOCAN estimates the incidence and mortality worldwide for 36 cancers in 185 countries and provides an update on cancer internationally [176]. A reported estimate is 19.3 million new cancer cases (18.1 million excluding non-melanoma skin cancer) and almost 10 million cancer deaths (9.9 million without non-melanoma skin cancer) occurring in 2020 worldwide. Female breast cancer has exceeded lung cancer as the most frequently diagnosed cancer, with an estimated 2.3 million new cases (11.7%), followed by lung (11.4%), prostate (7.3%), colorectal (10%), and stomach (5.6%) cancers [177]. For the effective treatment of breast cancer, surgery, chemotherapy, radiation therapy, hormonal therapy, and targeted therapy are performed [178]. However, nowadays, nanotechnology has gained interest for breast cancer treatment. Various organic and inorganic nanocarriers are used to deliver drugs to the specific target site [179]. Nanocarriers enhance the hydrophobicity of the anticancer drugs and promote specific target drug delivery [180]. Organic nanocarriers include polymeric nanocarriers, liposome nanocarriers, and solid lipid nanocarriers, while inorganic nanocarriers include magnetic nanocarriers, quantum dots, and carbon nanotubes (CNTs); both categories show great results towards treatment of heart diseases (Table 4) [181]. The mechanism of drug delivery in breast cancer is shown in Figure 4.

### 4.3. Lung Cancer

Lungs are basically responsible for inhalation [194]. The lung is composed airways (conveying the air inside and outside of the lungs) and alveoli (gas exchange zones) [195]. In fact, airways are comparatively tough barriers for particles to enter through, while the barrier along the alveolar wall and the capillaries is relatively fragile in the gas exchange component [196]. The huge exterior area of the alveoli and deep air blood exchange cause the alveoli to be less healthy when affected by environmental injuries. Such injuries may be the reason for some pulmonary illnesses, including lung malignancy [197]. Several nanoparticles are now being established for respiratory applications that aim at eliminating the restrictions of orthodox drugs [198] (Table 5). Nanoparticles aid the cure of many lung diseases, such as asthma, tuberculosis, emphysema, cystic fibrosis, and cancer [199].

## 5. Drug Delivery Approach in Heart Diseases

Cardiovascular diseases include myocardial infraction (MI) [213], ischemic impairment, coronary artery disease (CAD), heart arrhythmias, pericardial disease, cardiomyopathy (heart muscle disease), and congenital heart disease [214,215]. All these illnesses are the basic main cause of mortality and morbidity in the world [216]. Cardiac diseases in humans involve incongruity in the morphogenesis of heart arrangement, functionality, and the healing and periodic shrinkage of cardiac muscles [217,218]. Around 50% of patients suffering from MI die within five years [216]. The insistence for a novel and effective remedy has brought about progress in direct drug carriage to the heart [219]. Modern therapeutic approaches have been developed to stop the incidence of heart failure after myocardial infarction [220]. Liposomes, silica NPs, dendrimers, cerium oxide NPs, micelles, TiO_2_ NPs, stents with nano-coatings, microbubbles, and polymer–drug conjugates are used for drug delivery. Magnetic nanoparticles like magnetoliposomes (MLs) are made up of the union of liposomes and magnetic nanoparticles. They are used as magnetic-targeted drug delivery [221]. The PEGylation of MLs increases their rate of flow in the blood, and pairing of the MLs with antibodies raises the rate of active target to pretentious positions [222]. Namdari and his co-workers performed experiments in a mice model afflicted with myocardial infraction (MI). Liposomes are used with various modifications and in different ways; they are adapted to load drugs on NPs for efficient delivery inside the cell. Cationic liposomes, perfluorocarbon nanoparticles, polyelectrolyte nanoparticles, and polymeric nanoparticles are the modified forms of nanocarriers [223] (Table 6).

## 6. Drug Delivery Approach in Skin Diseases

Skin diseases are follicular and cutaneous. These dermatological diseases are treated nowadays with nanotechnology. Nanoparticle delivery for cutaneous disease treatment is preferred, with minor side effects. The conventionally used creams, gels, and ointments are insufficient for delivering drugs due to low penetration in skin tissues. To address this, polymeric, lipid, and surfactant nanocarriers are used. The polymeric micelles enhance drug penetration into the skin tissue to treat skin cancer. As in this reported study, chitosan polymeric NPs, liposomes, and gold nanoparticles can treat atopic dermatitis by improving drug penetration into the dermal and epidermal layers [246]. Gold nanoparticles are extremely small in size and can penetrate easily and effectively with very low toxicity and no skin damage. As such, they are used widely in nanocarrier formulations for skin diseases.

## 7. Drug Delivery Approach in Bone Diseases

Bone diseases includes bone defects due to many pathological factors, such as fracture, trauma, osteoporosis, arthritis, infections, and many other diseases. In fact, bone regeneration as a disease treatment is a very complex process, due to which nanomaterials and biological materials are fused to repair bones effectively. The combination of biomaterial and nanomaterial has reduced bone implantation through the development of bone bioscaffolds [247].

### Mechanism of Drug Delivery

**Figure 5:**
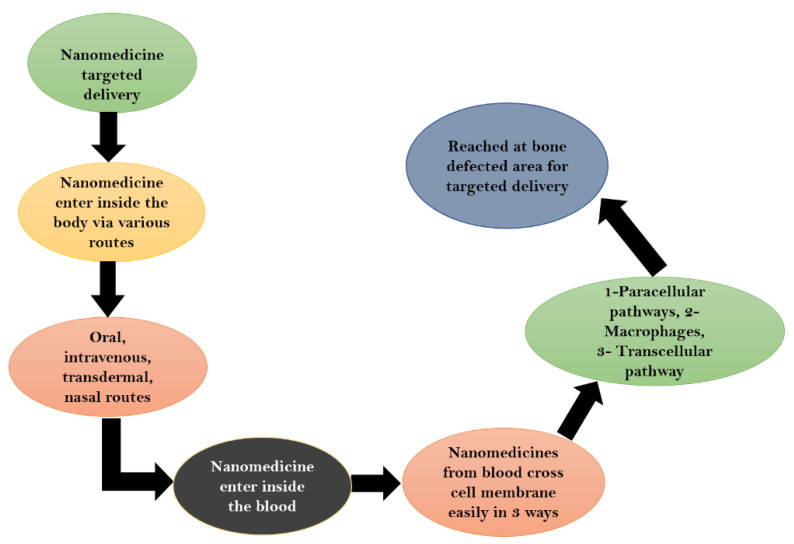
Mechanism of nanomedicine delivery in bone diseases.

The drugs encapsulated inside the nanoparticle is delivered through blood to the targeted area in the bones. The management of the sending nanoparticles as shown herenin (Figure 5).

## 8. Drug Delivery Approach in Blood Diseases

There are various types of blood diseases, like hemopoietic blood disorder, as well as iron deficiency, leukemia, anemia, hemophilia, platelet diseases, and blood cancer. The conventionally used chemotherapeutic system causes damage to the immune system, with high risk of mortality. Bone marrow transplant is also an expensive and intricate process. For example, thalassemia is treated with deferoxamine, a chelating agent to treat excessive iron in the blood. The siRNA-coated nanocomposite has the inhibitory activity for tumor cells in vivo [248]. The treatment of blood disorders with nanomedicine is still under investigation.

## 9. Future Challenges of Nanomedicines

In the field of nanomedicine, there are many innovations which show its importance in clinical and other medical aspects. Many scientists have investigated in their research how nanomedicine is involved in treating malignancies and reducing mortality and morbidity rates. However, there are also future challenges that nanomedicines have been facing until now [249]. The implementation of nanomedicine in clinical practice will face many issues with insurance companies, regulatory agencies, and the public health sector. Until now, the FDA has not developed any specific regulation for the products containing nanomaterials. Due to a lack of nanomaterial standardization and other safety issues, US agencies, such as the EPA and NIOSH, are giving less funding to these research endeavors.

## 10. Conclusions

Nanotechnology-based nanomedicine is a diverse field for disease treatment. Nowadays, in every sort of disease, nanotechnology is emerging as the best therapeutic to cure disease. At California University, researchers are developing methods to deliver cardiac stem cells to the heart. They attached nanovesicles that directly target injured tissue to increase the amount of stem cells there. Thus, the involvement of stem cells with nanotechnology will develop many solutions for the disease-based queries in the medical arena. However, nanomedicine and nano drugs deal with many doubts. Irregularities and toxicity and safety valuations will be the topic of development in the future. Nanotechnology will be in high demand. Nowadays, drug-targeted delivery through nanoparticles is catching the attention of pharmaceutical researchers all over the world. Nanomedicine will overcome all the side effects of traditional medicines. This nanoscale technology will be incorporated in the medical system to diagnose, transport therapeutic drugs, and detect cancer growth, according to the National Cancer Institute. Experts are trying to treat SARS-CoV-2 with nanomedicine, as nanoparticles with 10–200 nm size can detect, for site-specific transfer, SARS-CoV-2, exterminate it, and improve the immune system of the body. Nanotechnology could help to combat COVID-19 by stopping viral contamination. Highly accurate nano-based sensors will be made in the future that will quickly recognize the virus and act by spraying to protect frontline doctors and the public. Furthermore, many antiviral disinfectants are being developed through nanobiotechnology to stop virus dissemination. In the future, nanotechnology will evolve to develop drugs with high activity, less toxicity, and sustained release to target tissue. Therefore, personalized medicine and nanomedicine both will be potential therapies to treat COVID-19 successfully, as well as to treat upcoming diseases in future.

## Figures and Tables

**Table 1:** Evolution of nanoparticles from 1991 to 2022 in detail discussed here.

Year	Types of NPs	Drug Delivery Approaches	Diseases	Applications	Characterization	References
**1991**	Poly-alkyl-cyanoacrylate nanoparticles	Carrier that delivers drug to target specific site.	Cancer	Cancer chemotherapy and intracellular antibiotherapy.	Scanning electron microscope (SEM)	[37,38]
**1992**	Calcium hydroxyapatite ceramic (CHC)	Drug gentamicin placed in the porous blocks of calcium hydroxyapatite antibiotics (CHA).	Chronic osteomyelitis (animal model)	The bactericidal activity was retained and drug shows effective results.	No in vivo experiments performed	[39,40]
**1993**	Nano and micro particles	Micro-particulate system used for the administration of the drug.	Enhance oral immune system (immunization)	In vitro, self-diffusion, liberation due to erosion, pulsed delivery due to oscillating field.	In vitro experiments performed	[41,42]
**1994**	Acrylic acid copolymer NPs	Acrylic acid, acrylic amide, acrylic-butyl ester, and methacrylic methyl ester used as copolymer in drug delivery.	No	Help opsonin to reach specific target site and also enhance reticuloendothelial system.	Small angle X-ray scattering	[43,44]
**1995**	Poly-alkyl-cyanoacrylate (PECA) nanoparticles	Ofloxacin (OFX) and perfloxacine entrapped in PECA nanoparticles. OFX system more efficient than PFX system.	Bacterial diseases	The fluoro-quinolone-loaded nanoparticles enhance antimicrobial activity of the drug.	Freeze fracture electron microscopy, physicochemical characterization	[45,46]
**1996**	Protein and peptides-based NPs	Monoclonal antibodies, recombinant proteins transported to BBB by chimeric peptide approach.	Alzheimer’s disease	Avidin conjugate with BBB vector to transport all proteins across BBB. Vasoactive intestinal peptide cures brain diseases.	No characterization of physiologic-based strategy	[47,48]
**1997**	Nanoparticle	Nanoparticles as carrier to deliver drug to intra-arterial localization system. Cather based delivery	Restenosis (arterial reobstruction)	Easily penetrate into the arterial wall and without causing injury. Biocompatible and effective for restenosis treatment.	No	[49,50]
**1998**	Diblock copolymer nanoparticles	Micelles and nanosphere carry genes and hydrophobic drugs to target site.	No	Help to sustain drug rate. Solubilize, release, and protect drugs. Enhance retention time in the blood.	No	[51,52]
**1999**	Chitosan nanoparticles	Potential of chitosan nanoparticles to improve absorption of insulin through nasal cavity.	Diabetes	MicroAB assay used to determine insulin loading and release.	Zeta potential, laser doppler anemometry, photon correlation spectroscopy	[53,54]
**2000**	Liposome with hyperthermia as nanoparticles	Increased drug delivery to tumor. Hyperthermia helps liposome to work properly.	Ovarian carcinoma	Helpful in human cancer treatment.	Experiments performed	[55,56]
**2001**	PEGylated poly-cyano-acrylate nanoparticles	Efficient drug carrier to deliver therapeutic molecules in prion disease test.	Prion Diseases	Long retention time in blood as compared to non-PEGylated nanoparticles. Brain and spleen target tissues show uptake higher in scrapie-infected animals.	Experiments performed	[57,58]
**2002**	Transferrin mediated receptor endocytosis	Transferrin and transferrin receptor in drug and in gene transference via the BBB.	Cancer and Brain diseases	Transferrin receptor interceded iron uptake; regulation of transferrin receptor expression, anticancer drugs site-specific to tumor cells.	No	[59,60]
**2003**	L-nanoparticles	Intravenous injection of L-particles loaded with green dye shows hepatocellular carcinoma in humans.	I-Hepatitis B II-Hepatocellular carcinoma III-Hemophilia	Hepatitis B virus infects liver hepatocyte cells. L-nanoparticles deliver drugs or genes efficiently and specifically to the targeted hepatocyte cells in a mouse xenograft model.	No	[61,62]
**2004**	Colloidal gold nanoparticles	Colloidal gold nanoparticles used as vector to carry tumor necrosis factor (TNF) towards specific part of tumor in mice.	MC-38 carcinoma tumor	The designed vector PT-cAu-TNF bound on the surface of the gold NPs. Intravenous injection shows effective results in MC-38 carcinoma tumor.	TEM, dynamic light scatter, and differential centrifugal sedimentation, zeta potential	[63,64]
**2005**	Liposomes, nanoparticles	Vitamin Folic acid placed inside cationic liposomes and conjugate liposomes to folate ligand act as carrier and chemotherapeutics agents, and DNA attaches to the receptor-bearing cancer cells in vitro.	Cancer (human nasopharyngeal and prostate tumor)	Folate-associated, lipid-based nanoparticles transport DNA with high transfection efficacy and constraining tumor progress with intratumoral shot into human nasopharyngeal and prostate malignancy using an HSV-tk/GCV treatment system.	No	[65,66]
**2006**	Folate-conjugated starch nanoparticles (StNP’s)	Folate changed with PEG coupled to the exterior of starch NPs to attain the FA-PEG/StNPs. Doxorubicin loaded on FA-PEG/StNP.	Liver cancer	In vitro, FA-PEG/StNP targeted on liver cells BEL7404. It reduced DOX toxicity. This combination can be suitable for cancer targeting drug haulers in future.	AFM and zeta potential, UV Spectro-photometer characterize particle size determination	[67,68]
**2007**	Gold nanoparticles (AuNPs)	Drug and gene delivery approach to deliver drugs and genes by using gold nanoparticles. The transfection efficacy for beta galactosidase with various MMPCs.	Human nasopharyngeal carcinoma	Properties of drug transfer like reduced toxicity, treating acute diseases, uptake and release rate using fluorophore AuNPs provide added insight in future.	Fluorescence and bright-field microscopy	[69,70]
**2008**	PEGylated gold nanoparticles	Very effective drug transfers with AuNPs’ vector for in vivo photodynamic treatment in cancer.	Cancer	The diversity in medicine released in vitro in two-phase solution system. In vivo in cancer-bearing mice shows that the way of drug carriage is enormously well-planned, and submissive targeting prefers the tumor area.	TEM and image analysis, DLS measurement, UV-vis, and fluorescent spectrophotometer	[71,72]
**2009**	Alginate/ Chitosan (Alg/Chi) nanoparticles	Nanoparticles of alginate/chitosan polymers were arranged by pre-gel preparation method via drop-wise addition of several concentrations of CaCl_2_ to a definite concentration of sodium alginate.	No	Optimization of Alg/Chi NPs and preparation are areas of this research. Some parameters like ratio of Alg/Chi, ratio of CaCl_2_/Alginate and N/P can disturb size and loading ability of these particles.	Zeta potential, photon correlation spectroscopy, scattering particle size analyzer, FTIR analysis, DSC analysis	[73,74]
**2010**	Mesoporous silica nanoparticles	Targeted carriage of chemotherapeutic mediator methotrexate (MTX) to tumor cells by means of poly (ethylene mine)-functionalized mesoporous silica small units as vectors for drug delivery.	Cancer	(a) Choice of adaptable surface functionalization; (b) High level of cell specificity and effective cellular uptake; (c) A slight grade of early seepage and the measured release of the medicine; (d) Low cytotoxicity of the transporter.	Scanning electron microscope (SEM)	[75,76]
**2011**	Nano diamond (ND) or diamond nanoparticles	Nano diamonds have ability to transport small interfering RNA into sarcoma (Ewing) cells. Was examined with evaluation of the route of in vivo anticancer nucleic acid drug transfer.	Ewing Sarcoma Cells (Cancer)	Well-organized delivery of oligonucleotide by a cationic nano-diamond nanoparticle: (i) Suitably robust adsorption of the biomolecule on the particle surface across the cell membrane deprived of damage of material; (ii) The severance of the compound on the time-scale of a cell division cycle.	FT-IR confirm the absorption of PAH on nano-diamonds and zeta potential	[77,78]
**2012**	Silver nanoparticles	This method was to design stable silver NP vector to make larvicides of mosquitos to destroy mosquitos’ life with drugs.	Malaria, Dengue fever, Filariasis	The leaf potage of *Annona squamosa* used as an active capping and reducing mediator for the fusion of silver nanoparticles.	Ultraviolet spectrophotometry, X-Ray diffraction, FT-IR, SEM	[79,80]
**2013**	Silver nanoparticle	Nanoparticles of noble metal show potential as photo-activated vectors for drug delivery. SNPs conjugated with thiol-terminated photo-liable DNA oligonucleotides.	Photo-activated gene silencing	Good consistency to nucleases, hybridization amplified action upon photo release, and effective cellular uptake as associated to commercial transfection vectors.	UV-spectrophotometer, fluorescent confocal microscopy	[81,82]
**2014**	Silver nanoparticles as drug-loading vector	Silver nanoparticles synthesized from plant *Pongamia pinnata* by green method.	Dengue	Medically active plant and earth eco-friendly. Larvicidal action of silver nanoparticles and leaf extract contrary to *Aedes aegypti* showed positive results.	UV-visible absorption spectrum, TEM, XRD, FTIR	[83,84]
**2015**	Polyamidoamine nanoparticles	Polyamidoamine nanoparticles work as nanocarrier and deliver anti-malarial drug to the targeted sites. It also works as nanomedicine.	Malaria	Union of doxorubicin and polymers increases drug solubility, enhances its blood half-life, decreases toxicity, and enhances targeting.	Fluorescence-assisted cell sorting, transmission electron microscopy, confocal immunofluorescence	[85,86]
**2016**	Solid Lipid nanoparticles (SLNP)	Electroporation and nanocarrier used to deliver drugs. In this study, SLNP laden with cyanine type IR-780, flavonoid derivatives, photosensitizer through solvent diffusion method.	Colon cancer	Drug transfer potential of therapeutics compressed with electroporation.	Confocal laser scanning microscopy (CLMS) for the estimation of F-actin AFM and DLS	[87,88]
**2017**	Filamentous bacteriophage and phage-mimetic nanoparticles	Delivery of drug and gene through phage particles. Phage can be chemically altered or genetically designed to load drugs and transfer foreign genes.	Bacterial and viral diseases	Filamentous bacteriophage used in the making of mark medicine transfer as virus-based delivery system. The bacteriophage uncovered with mark-definite peptides or antibodies can be bound with other carriers (such as liposomes, inorganic NPs) to make a unique transfer scheme.	No	[89,90]
**2018**	Mesoporous silica nanoparticles (MSNs)	Through electrostatic absorption, MSNs loaded with surface-hyper-branching polymerized poly (ethylene- mine) for loading siRNA.	No	The practice of non-viral vectors can solve most of these problems like short time, noxiousness while inorganic, and non-viral vectors, like MSNs, are also very affordable and vigorous.	Transmission electron microscopy, dynamic light scattering (DLS), and zeta potential involved in particle size determination	[91,92]
**2019**	Chitosan nanoparticles	Drug loaded on chitosan nanoparticles to deliver to targeting sites. All types of drug delivery sites involved.	No	Ocular drug delivery, vaccine delivery, perioral delivery, vaccine transfer, mucosal and nasal drug transfer, gene carriage, pulmonary drug delivery, buccal medicine distribution, vaccine transfer, and cancer treatment.	No	[93,94]
**2020**	Mesoporous silica NPs with folic acid (MSN−COOH-Tet-HBP-FA)	This approach is pH subtle drug delivery system built on folic-acid-targeted HBP to re-form/reshape the mesoporous silica nanoparticles.	Cancer	The hyper-branched polymer HBP encapsulates the drug particles in the mesopores as a lid, which progresses the permanency of the carrier material and permits the drug to attain “zero pre-release” within 20 h in a usual physiological atmosphere.	XRD, TEM, HNMR spectra, SEM, UV-analysis, Thermogravimetric analysis (TGA)	[95,96]
**2021**	Novel silver nanoparticles	In this approach, DNA or messenger RNA (mRNA) sequences are transported to the body to produce proteins, which copy disease antigens to arouse the immune response.	SARS-CoV-2	The nucleic acid vaccines comprise cell-mediated and humoral immunity activation, affluence of strategy, quick malleability to altering pathogen strains, and customizable multi-antigen vaccines. To fight the SARS-CoV-2 epidemic and many other ailments, nucleic acid vaccines seem to be a hopeful way.	No	[97]
**2021**	1-Lipid based nanoparticles 2-Metal and metal oxide NPs 3-Resveratrol-zinc NPs	These nanoparticles have crucial role in the COVID-19 success rate. Metals such as Au, Ag, Zn, Cu have potential in controlling coronavirus due to their discrete features. It is a drug delivered via carrier. It gives immuno-anti-inflammatory viral retort.	COVID-19 SARS-Cov-2 viral disease COVID-19	It helped in the COVID-19 treatment vaccines, such as Doxil and Onpattro, and has a good success rate. Such NPs have been used in prevention like face masks, various immune sensors, and coatings on various things. Resveratrol-zinc nanoparticles possess a chief pharmacokinetic gain for COVID-19.	No COVID-19 mono and adjuvant therapy	[98,99,100]
**2022**	1-Iridium oxide NPs 2-Chitosan nanoparticles	A nanoprobe was synthesized for in vivo fluorescence tomography of microRNA and coactive photothermal dealings of lump. It is a biotic macromolecule-based medicine transfer system to advance the curative potential of non-natural neural control networks.	Cancer Nervous breakdown	Nanoprobe helped in vivo in healing studies and continuously killed the lump growth. Theses neuroprotective mediators are merged into the structure of NGCs and delivered into brain via NPs.	No Nanocarriers are biocompatible, biodegradable, non-immunogenic, constant, and hold tunable properties	[101,102]

**Table 2:** Advantages and disadvantages of nanomedicine.

Nanomedicine Names	Advantages	Disadvantages	Ref.
***Tacrine*** **-loaded polymeric NPs**	NPs are reserved in the brain for long time, biocompatible, low in cost, control drug release, and targeted conjugation with ligands	Slowly degradable, sometimes uncertain toxicity	[145]
***Rivastigmine*** **-loaded polymeric NPs**	They increase drug concentration in the brain, avoid phagocytosis by RES	Increase oxidative stress, toxicity	[146]
***Piperine*** **-loaded SLNPs**	Widely examined, fewer side effects of drugs, improved therapeutic effects and drug solubility	Low loading capacity, easily cleared by reticuloendothelial system	[147]
***Folic-acid*** **-loaded liposomes**	Highly biocompatible and biodegradable, High stability and bioavailability, active surface targeted	Difficulty in binding with lipids, low stability and drug carriage rate	[148]
***Beta-Asarone*** **-loaded nanoemulsions**	Improved bioavailability, capability to hydrolyze hydrophobic and hydrophilic drugs	Thermodynamically unstable, instant drug release	[149]

**Table 3:** Various nanoparticles involved in brain cancer treatment in recent era.

NP Name	NP Types	Drug Loaded on NPs	Cancer Type	Model	Action	Ref.
DOX-SL-GG AuNPs	Gold nanoparticles	Doxorubicin	Glioma and glioma stem cell lines	In vitro	Endocytosis occurs. Cytotoxic activity increased both on LN-229 glioma cells and HNGC-2 glioma stem cells.	[157,158]
Lapatinib-loaded human serum albumin	Albumin-bound nanoparticle	Lapatinib	Brain metastasis	Murine model in vitro	Constrain movement, invasion and adhesion of high brain-metastatic 4T1 cells.	[159,160]
Lapatinib-incorporated lipoprotein like NPs	Lipoprotein-like nanoparticles	Lapatinib	Glioma	In vivo murine model	Both LTNPs (10 mg kg^−1^) and LTNPs (30 mg kg^−1^) significantly constrain the progress of U87 xenografts.	[161,162]
Gold–iron oxide nanocomposites	Curcumin–lipoic acid conjugate	Glutathione	Brain cancer	Cytotoxicity and apoptosis assay	Comparatively greater cytotoxicity against cancerous U87MG cells than standard astrocyte cells.	[163,164]
Tocopherol polyethylene glycol chitosan nanoparticles	Fabricated synergistic bioadhesive nanoparticles	Docetaxel	Brain cancer	Enhance cellular uptake and cytotoxicity	Synergistic influence of nanoparticles has increased the delivery of docetaxel into brain melanoma cells.	[165,166]
Chitosan or glycol chitosan (GCS) nanoparticles (NPs)	Methotrexate-loaded chitosan and glycol chitosan-based nanoparticles	Methotrexate (MTX)	C6 glioma cells	Cytotoxicity assay and cell lines	Nanoparticles show cytotoxicity against C6 cells line and are able to control MDCKII-MDR1 cell hindrance.	[167,168]
Lipid–drug-conjugated (LDC) nanoparticle	5-FU (fluorouracil)nanoparticles	Fluorouracil	Brain cancer glioma cells	In vitro cytotoxic activity and human glioma cell lines in vivo	The effectiveness of 5-FU to medicate the brain malignancy is improved when it is designed with LDC nanoparticles.	[169,170]

**Table 4:** Nanoparticles’ role in treatment of breast cancer.

Nanomaterial (Organic Nanomaterial)	Material Used	Drug Loaded with NPs	Animal Model	Disease	Description	Ref.
Solid lipid nanoparticles (SLNPs)	Folic-acid-receptor-targeted solid lipid nanoparticles	Letrozol (LTZ) Folic acid	In-vitro MCF-7 cancer cell lines	Breast cancer	Lactate dehydrogenase (LDH) and 3-(4, 5-Dimethylthiazol-2-yl)-2, 5-diphenyltetrazolium bromide (MTT) assays to check cell membrane damage. Caspase-3 activity and TUNEL assays were performed to confirm induced apoptosis.	[182,183]
Curcumin–Solid Lipid nanoparticles (CURC-SLNs)	CURC-loaded SLNs and doxorubicin p-glycoprotein (Pgp)	Doxorubicin (DOX)	In-vitro	Breast cancer	Curcumin-loaded SLNs 5–10 folds more effectively than curcumin in free form, increasing toxicity in Pgp-expressing triple negative breast cancer.	[184,185]
Copolymer-magnetite nanoparticles	doxorubicin–core-shell chitosan nanoparticles	Doxorubicin (DOX)	In-vitro	HER2-over-express in breast cancer	Anti-HER2-conjugated O-succinyl chitosan graft pluronic F127 copolymer nanoparticles are effective for the making of anticancer drug carriers.	[186,187]
Polymeric nanoparticles	PEGylated ε-poly-l-lysine polymeric nanoparticle	doxorubicin and lapatinib	In-vitro	MCF-7 breast cancer cell	Combination remedy by DMMA-P-DOX/LAP nanoparticles constrains the solid tumors to shrink or disappear completely in the MCF-7 tumor model.	[188,189]
**Nanomaterial (Inorganic Nanomaterial)**	**Material Used**	**Drug Loaded on NPs**	**Animal Model**	**Disease**	**Description**	**Ref.**
Colloidal gold nanoparticles Iron-based metal network	Gemcitabine-hydrochloride (GEM)-loaded colloidal gold nanoparticles	Gemcitabine	In vitro (MDA-MB-231) cell line	Human breast cancer adenocarcinoma	Gemcitabine-hydrochloride-loaded gold nanoparticles developed using gum acacia as a polysaccharides-based system.	[190,191]
Magnetic nanoparticles	L-carnosine-coated magnetic nanoparticles (CCMNPs)	L-carnosine	In vitro In vivo	Breast cancer	CCMNPs were targeted precisely, amassed in lump, showing noteworthy decrease in lump mass size with no general harmfulness.	[192,193]

**Table 5:** Recent discovered nanoparticle’s role in lung cancer treatment.

Nanoparticles	Exposure Method	Animal Model	Description	Used for	Reference
Poly (L-aspartic acid co lactic acid)/DPPE copolymer nanoparticles	Intraperitoneal injection	Mouse xenograft model	DPPE co-polymer NPs laden with doxorubicin (DOX)	Lung melanoma	[200,201]
Poly (β-amino ester) nanoparticle (PBAE)	Intratumoral injection	Mouse xenograft model	PBAE polymers that self-assemble with DNA and evaluated for transfection effectiveness in the p53 mutant H446 SCLC cell line	Small cell lung cancer	[202,203]
Lipid polymeric nanoparticles	Intraperitoneal injection	Mice	The receptor factor (EGF) was co-designed with cisplatin plus doxorubicin	Lung carcinoma	[204]
Doxorubicin and cisplatin (CDDP) co-loaded nanoparticles	Pulmonary administration	Mouse model	Methoxy poly -poly (ethylenimine)-poly(l-glutamate) copolymers were manufactured as a transporter for the codelivery of DOX and CDDP	Metastatic lung melanoma	[205,206]
Redox-responsive plus pH-sensitive nanoparticles	Subcutaneous injection	Mouse xenograft model	PAA-ss-OA-modified Erlotinib (ETB)-loaded lipid nanoparticles (PAA-ETB-NPs) were made using the emulsification and solvent evaporation method	Non-small cell lung melanoma (NSCLC)	[207]
Nanoparticles/mesenchymal stem cell (MSC)	Injected by loading on NPs inside the body	Rabbit, mice, and monkey	MSC as lung-melanoma-targeted drug transfer transporters by loading nanoparticles (NPs) with anticancer medicine. MSC demonstrated a greater medicine ingestion ability than fibroblasts	Lung melanoma	[208,209]
Hyaluronic-acid-based lipid nanoparticle	Dialysis techniques used in in vitro study	No	Assessment of the capacity of hyaluronic-acid-based nanostructured lipid carriers (NLCs) to improve apigenin (APG) efficacy as Nrf2 inhibitor, in immediate administration with DTX in A549 NSCLC	Lung cancer	[210]
MAGE-A_3_ NIR insistent luminescence nanoparticles	In vitro activity	In vivo mouse model	Cancer-definite hybrid theranostics nanomaterials MAGE-A3 NIR insistent glow nanoparticles coupled to Afatinib for in situ conquest of lung adenocarcinoma	Non-small cell lung carcinoma	[211]
Hyaluronic-acid-based nanoparticle	In vivo In vitro	Mice used; in vitro assays used	Paclitaxel delivered via these NPs to cancerous cells to reduce or stop drug confrontation	Carcinoma	[212]

**Table 6:** Different forms of NPs; their experiment studies show its role in treatment of heart diseases.

Nanocarriers	Experimental Model	Agents	Results	References
Polymeric (PLGA) nanoparticle	Balloon injured carotid and stented porcine coronary artery in rats	AG-1295 and AGL-2043	Inhibition of restenosis	[224,225,226,227,228,229,230]
Perfluorocarbon nanoparticles	Human plasma lumps, hyperlipidemic animals	a3b integrins, surface-bound streptokinase, others	In vitro fibrinolysis and in vivo theranostics	[231,232,233,234,235]
Cationic nanoparticles	Clinical test, patients with 60 to 99% stricture in main arteries, confined supply via catheter (tube)	Vascular endothelial growth factor is involved to encode viral vector	Major improvement in myocardial perfusion	[236,237,238,239,240,241]
VEGF nanoparticles	Mice, murine myocardial infarction model	VEGF proangiogenic cytokine	Myocardial perfusion in coronary patients for heart repair	[242,243,244,245]

## Data Availability

Not applicable.
